# Effect of a continuity of midwifery care model that used a respectful maternal care framework in Korea: a non-randomized study

**DOI:** 10.3389/fpubh.2025.1578158

**Published:** 2025-05-07

**Authors:** Geumhee Jeong, Hyun Kyoung Kim, Uri Bang

**Affiliations:** ^1^Department of Nursing, Hallym University, Chuncheon, Republic of Korea; ^2^Department of Nursing, Kongju National University, Gongju, Republic of Korea

**Keywords:** maternal health services, midwifery, pregnant women, prenatal education, respect

## Abstract

**Aim:**

This study evaluated “Team-Mamas,” a continuity of midwifery care intervention that used a respectful maternity care framework during the antenatal, labor, and postpartum periods. The research aimed to assess the impact of intervention on birth outcomes, birth satisfaction, birth experience, and maternal function.

**Participants and setting:**

This study employed a non-equivalent control group post-test design. The midwife companion program offered services including natural childbirth education, prenatal healthcare, birth rehearsal, childbirth support, infant care, and postpartum education and counseling. This program provided continuous support by midwives from the 28th week of gestation until 14 days after birth. There were 65 participants from 3 cities in Korea from March to November, 2023.

**Results:**

The intervention led to lower frequencies of episiotomy (*p* < 0.001), oxytocin augmentation (*p* = 0.005), epidural anesthesia (*p* = 0.007), and analgesic use (*p* < 0.001), as well as higher breastfeeding rates at 1 week (*p* = 0.012) and 4 weeks (*p* = 0.004) postpartum in the experimental group compared to the control group. Both birth satisfaction (*p* < 0.001) and birth experience (*p* < 0.001) scores were higher in the experimental group compared to the control group. No statistically significant differences were found between the two groups regarding neonatal birth weight (*p* = 0.346) and maternal function (*p* = 0.067).

**Conclusion:**

Mothers experienced satisfactory and safe birth outcomes when supported by a continuity of midwifery care intervention. We suggest promoting positive birth outcomes and experiences through integrated support that honors the dignity of mothers throughout pregnancy, labor, birth, and the postpartum period.

**Clinical trial registration:**

Identifier (KCT0008956) in the Korean Clinical Research Information Service.

## Background

Continuous care during childbirth can have positive effects on both the mother and the newborn. Continuity of midwifery care (CMC) models are designed to support women by having a midwife serve as the primary care provider in community or hospital settings (1). CMCs involve one or a small team of midwife providing care during pregnancy and birth, extending into the postnatal period (2). CMC supports pregnancy and birth as transformative experiences through holistic care with minimal interventions (1). Building a relationship between women and midwives resulted in safe, secure, trusting, confident, and respectful experiences (3). A recent meta-analysis (1, 2, 4) and realistic review (3) regarding CMC reported that close attention to women’s individual needs by midwives increased the frequency of vaginal spontaneous delivery and decreased rates of cesarean section, episiotomy, local analgesia, vacuum delivery, and admission to neonatal intensive unit. The CMC philosophy contains normalizing, humanizing, and respectful care for women (3, 4).

The World Health Organization (WHO) presented guidance on respectful maternity care (RMC) for avoiding of unnecessary interventions that would negatively impact maternal and fetal wellbeing (5), and CMC antenatal care has been recommended in settings with well-functioning midwifery programs (6). One of the most important principles of RMC is continuous support from a skilled maternal care provider (5). The International Confederation of Midwives (ICM) supports midwives as the preferred care providers for childbearing women globally, advocating for a care model rooted in respect, compassion, human rights, and a guiding midwifery philosophy (7). Therefore, CMC adopts the concepts of RMC which maintains maternal dignity, privacy, and confidentiality to offer continuous support (5). The CMC approach helps pregnant women feel respected and empowered, improving birth satisfaction (1, 2, 8). A medicalized model of maternity care, characterized by excessive interventions, has been shown to undermine women’s confidence in their ability to give birth and have a negative impact on their health. This was reported in a study that examined unsatisfactory birth experiences among Turkish women (9). An Ethiopian study found that only 35.8% of women received RMC, and the rights of only 39.3% of women to have their preferences respected were upheld (10). In Sweden, factors such as physical distress, disrespectful behavior from partners and caregivers, and inadequate facilities contributed to negative birth experiences (11). In South Korea, as the demand for maternal initiative in labor has grown, midwifery care has been shown to alleviate discomfort from physical, environmental, social, and cultural factors, resulting in higher satisfaction with care services compared to hospital deliveries (12). However, there is little research on the impact of CMC interventions on pregnant women’s experiences of childbirth.

The CMC intervention using RMC framework integrates key concepts such as consent, which relates to decisions about care, procedures, interventions, and autonomy, with an emphasis on participants’ choice and the importance of collaborative decision-making. These critical elements shape care decisions and have a major impact on outcomes, including maternal and neonatal health, as well as on women’s overall experiences, which constitute a central factor in determining clinical outcomes (13).

This study aimed to develop and test a CMC intervention for prenatal, birth, and postnatal women based on the RMC framework (5). The “Team-Mamas” CMC program was designed to enhance the quality of care for women and neonates, with a focus on woman-centered care based on a holistic, human rights-based approach. The specific objectives of this study were to evaluate the impact of the CMC program on: (1) maternal birth outcomes; (2) neonatal birth outcomes; (3) birth satisfaction, birth experience, and maternal function.

The hypotheses of this study were as follows:

*Hypothesis 1*: The experimental group participating in the CMC program will exhibit lower rates of episiotomy, analgesics, epidural anesthesia, and oxytocin augmentation than the control group.

*Hypothesis 2*: The experimental group participating in the CMC program will exhibit greater neonatal weight and a higher rate of breastfeeding than the control group.

*Hypothesis 3*: The experimental group participating in the CMC program will exhibit higher birth satisfaction, birth experience, and maternal function levels than the control group.

## Methods

### Study design

A non-equivalent control group, quasi-experimental post-test design was adopted to evaluate the effectiveness of a CMC program during pregnancy, childbirth, and the postpartum period according to the RMC framework (Supplementary material 1). This study adhered to the Transparent Reporting of Evaluations with Non-randomized Designs (TREND) reporting guidelines.

### Inclusion and exclusion criteria

The inclusion criteria for the study were as follows: (1) pregnant women who were over 28 gestational weeks, (2) those who expressed a desire to participate in the prenatal program, (3) participants who attended the full 10-week educational course, and (4) those who intended to have a normal vaginal spontaneous delivery. The exclusion criteria were: (1) pregnant women experiencing maternal or fetal health complications related to the current pregnancy, such as gestational hypertension, risk of miscarriage, or preterm labor, (2) women with maternal or fetal health issues due to conditions unrelated to the current pregnancy, including diabetes, kidney disease, or liver disease, (3) those anticipating a cesarean section, and (4) women who lacked proficiency in Korean. These exclusion criteria were chosen based on prior research on the impact of such factors on midwifery outcomes (14).

### Allocation

Convenience sampling was conducted among attendees of prenatal programs at four obstetric hospitals in Seoul, as well as two public health centers in Chuncheon and Gongju, South Korea from March to November, 2023. The researchers obtained permission to conduct the prenatal program from the managers of the hospitals and maternal centers. Pregnant women attending the antenatal education programs at these hospitals and health centers were recruited through announcements on the homepage and through leaflets. The allocation of participants to the experimental and control groups was not randomized, and the recruitment process was not blinded. The participants’ records from the births were collected, and online questionnaires were distributed to the participants 4 weeks postpartum using a Naver survey form. The experimental group consisted of individuals recruited from three hospitals in Seoul that were participated in the midwife companion program education. The control group was recruited from one hospital and two public health centers that offer prenatal childbirth education in Seoul, Chuncheon and Gongju.

### Intervention

**Figure 1:**
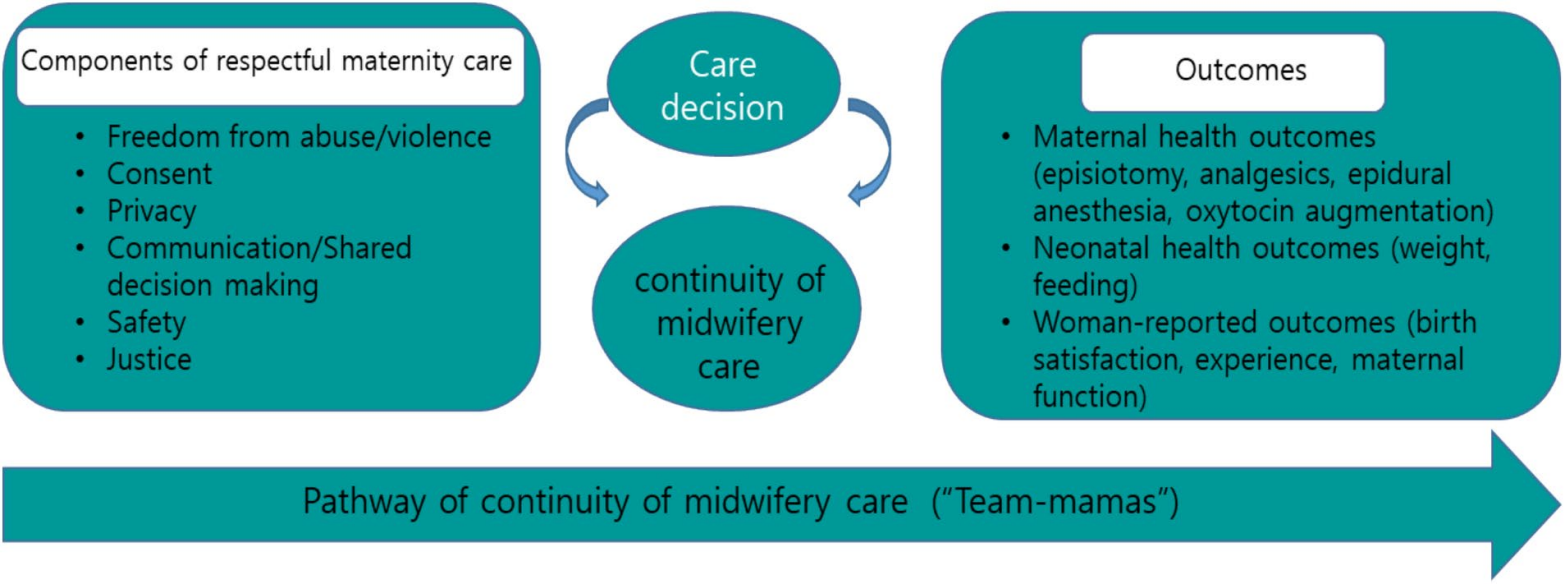
Conceptual framework according to continuity of midwifery care using respectful maternity care.

The researchers developed the content for the CMC program, known as “Team-Mamas,” based on the RMC framework (5). The CMC intervention was structured into 7 sessions, each employing personal education, consultation, counseling, and support methods to facilitate effective communication between midwives and women. “Team-Mamas” adopted a holistic, human rights-based approach, aiming to empower women to make informed and autonomous decisions about their childbirth experiences. In sessions 1 and 2, the “companion during pregnancy” component provided prenatal education and consultation from 28 to 32 gestational weeks. These sessions covered program orientation, natural childbirth, *taegyo* (a Korean traditional prenatal health care practice), and strategies for promoting prenatal health. Sessions 3 and 4, conducted between 34 and 37 gestational weeks, were dedicated to education about childbirth and postpartum preparation, with an emphasis on self-decision-making. Session 3 included a “birth rehearsal,” an intervention designed to prepare women for childbirth by educating them about the birthing process and assisting with planning for their desired childbirth experience. Session 4, titled “becoming a mom,” prepared women for postpartum care, helping them to envision their roles as parents and to plan for breastfeeding and baby care. Session 5, “companion during birth,” provided an intervention at the time of birth through midwife-led hospital delivery, offering physical and psychological support during childbirth and postpartum care within the first 3 h after delivery. Sessions 6 and 7, “companion during postpartum,” involved interventions to support breastfeeding, conduct neonatal health assessments, and facilitate postpartum adaptation during the first 1–2 days after birth. Session 7 was conducted daily for 10 min from postpartum day 3 to 14 via telephone and KakaoTalk, a Korean messaging application. The content of these sessions included consultations on neonatal check-ups, postpartum recovery, baby care, baby-parent interaction, and CMC wrap-ups. A post-test survey was administered 4 weeks following delivery (Figure 1).

To validate the content of the “Team-Mamas” intervention, it was assessed by three women’s health professors, one obstetrician, and three midwives for appropriateness, sufficiency, effectiveness, and feasibility. Discussions with participants about obtaining consent for natural childbirth, kangaroo care, breastfeeding, and other preferences were integral to the CMC program. The program spanned 10 weeks and included seven sessions, averaging approximately 8 h in total, in addition to the birth period, which ranged from 4 to 28 h. The CMC program comprised 6 face-to-face interventions and one remote intervention, all conducted by midwives from March to November 2023 (Table 1).

**Table 1:** “Team-mamas” continuity of midwifery care program.

Session	Time	Categories	Contents	Duration	Educator
1	28–32 gestational week	Prenatal education and consultation	- Orientation - Natural birth education -“Taegyo”: Korean traditional birth education - Diet and exercise during pregnancy	90 min	Midwife
2	32 gestational week	Telephone consultation	Health promotion during pregnancy Question and answer	30 min	Midwife
3	35–37 gestational week	Prenatal education and consultation	Birth preparation Breast feeding Neonatal care management	90 min	Midwife
4	37–41 gestational week	Birth education and consultation	Induction labor consultation Understanding of childbirth	30 min	Midwife
5	40 gestational week	Telephone consultation	Diet and exercise consultation Baby position assessment Breech position change exercise	30 min	Midwife
6	37–41 gestational week	Prenatal education	Exercise consultation Education birth symptom Rapport with doula	60 min	Doula (Standby birth 24 h)
7	28–41 gestational week	Additional telephone consultation	Consultation after every prenatal screening test Consultation in every high risk symptom	30–90 min	Midwife
8	At birth and 2–3 h after birth	Support birth	Hospital delivery assisted doula Physical support: pain, relaxation, breath, push, position, & massage Psychological support Postpartum care: assessment hemorrhage, fundus, and breastfeeding	1–2 day	Doula
9	Postpartum within 1 h	Postpartum consultation	Empowering breastfeeding - Neonatal health assessment	60 min	Midwife
10	Postpartum within 10 days	Postpartum telephone consultation	Everyday neonatal check-up Neonatal weight and dehydration check-up Wrap-up of the program: expression of birth process	100–120 min	Midwife

Participants in the control group received standard prenatal care through face-to-face interventions provided by health professionals, including a nursing professor, a nutritionist, and a breastfeeding expert. This intervention totaled 8 h, spread over four sessions of 2 h each, which took place in March, June, September, and November 2023 at the health centers. The program for the control group was structured as follows: Session 1 included an orientation, an introduction to *taegyo*, and prenatal exercise. Session 2 covered relaxation techniques, breathing exercises, and breastfeeding. Session 3 focused on understanding childbirth, pain control methods, and massage. Session 4 addressed postpartum care, prevention of postpartum blues, and neonatal care, and concluded with a wrap-up.

The post-test for both groups was administered 4 weeks after childbirth using an online questionnaire conducted by researchers. This questionnaire assessed maternal and neonatal outcomes, birth satisfaction, experience, and maternal function, and took approximately 10–15 min to complete. Upon completion of the survey, participants received an online gift valued at approximately 15 dollars.

### Measurements

#### Maternal birth outcomes

Maternal birth outcomes were assessed using a self-reported questionnaire 4 weeks after birth. This questionnaire included items on the treatment of episiotomy, use of analgesics, epidural anesthesia, and oxytocin augmentation during childbirth. The response options were 1 = yes, 2 = no, and 3 = unknown.

#### Neonatal birth outcomes

The birth outcomes of the neonates were evaluated using a self-reported questionnaire, which recorded the neonates’ weight, breastfeeding status at 1 week, and breastfeeding status at 4 weeks postpartum. The weight of the neonates was measured at birth in grams. The feeding options were 1 = breastfeeding, 2 = bottle-feeding, and 3 = mixed feeding.

#### Birth satisfaction

The Birth of Satisfaction Scale-Revised (BSS-R), developed by Martin and Martin (15), was utilized with permission. In this study, we employed the validated Korean version of the BSS-R to assess maternal satisfaction during childbirth (16). The BSS-R is composed of 3 dimensions: “quality of care provision” with 4 questions, “women’s personal attributes” with 2 items, and “stress experienced during labor” with 4 items. The scale includes a total of 10 questions, each rated on a 5-point Likert scale ranging from 1 (“not at all”) to 5 (“very much”). A higher score indicates greater satisfaction with the childbirth experience, with the total score ranging from 10 to 50. The internal consistency reliability Cronbach’s alpha was 0.70 in the original study, and 0.76 in this study.

#### Birth experience

The QUOTE-birth instrument, developed by Jeong et al. (17), was utilized to measure the birth experience after permission was granted for its use. The QUOTE-birth is composed of 4 factors: 10 items on family care, eight on personal care, five on affective empowerment, and five on information provision, totaling 28 questions. The responses for the QUOTE-Birth items are measured on a dichotomous scale, with 1 indicating “not performed” and 2 indicating “performed.” A higher score reflects a higher quality of delivery care, with the total score ranging from 28 to 56. Cronbach’s alpha was 0.96 in the original study and 0.77 in this study.

#### Maternal function

The Barkin Index of Maternal Functioning (BIMF) was used to assess maternal functioning in postpartum women (18). A validated Korean version of the BIMF, provided by the University of Pittsburgh, was employed in our research. The BIMF consists of 7 factors: self-care (3 items), infant care (2 items), mother–child interaction (3 items), psychological wellbeing (10 items), social support (3 items), management (6 items), and adjustment (2 items). Each BIMF item is rated on a 7-point Likert scale, ranging from 0 (“never agree”) to 6 (“strongly agree”), with higher scores indicating better maternal functioning. The overall score can range from 0 to 174. Cronbach’s alpha was 0.87 in the original study and 0.89 in this study.

### Sample size

**Figure 2:**
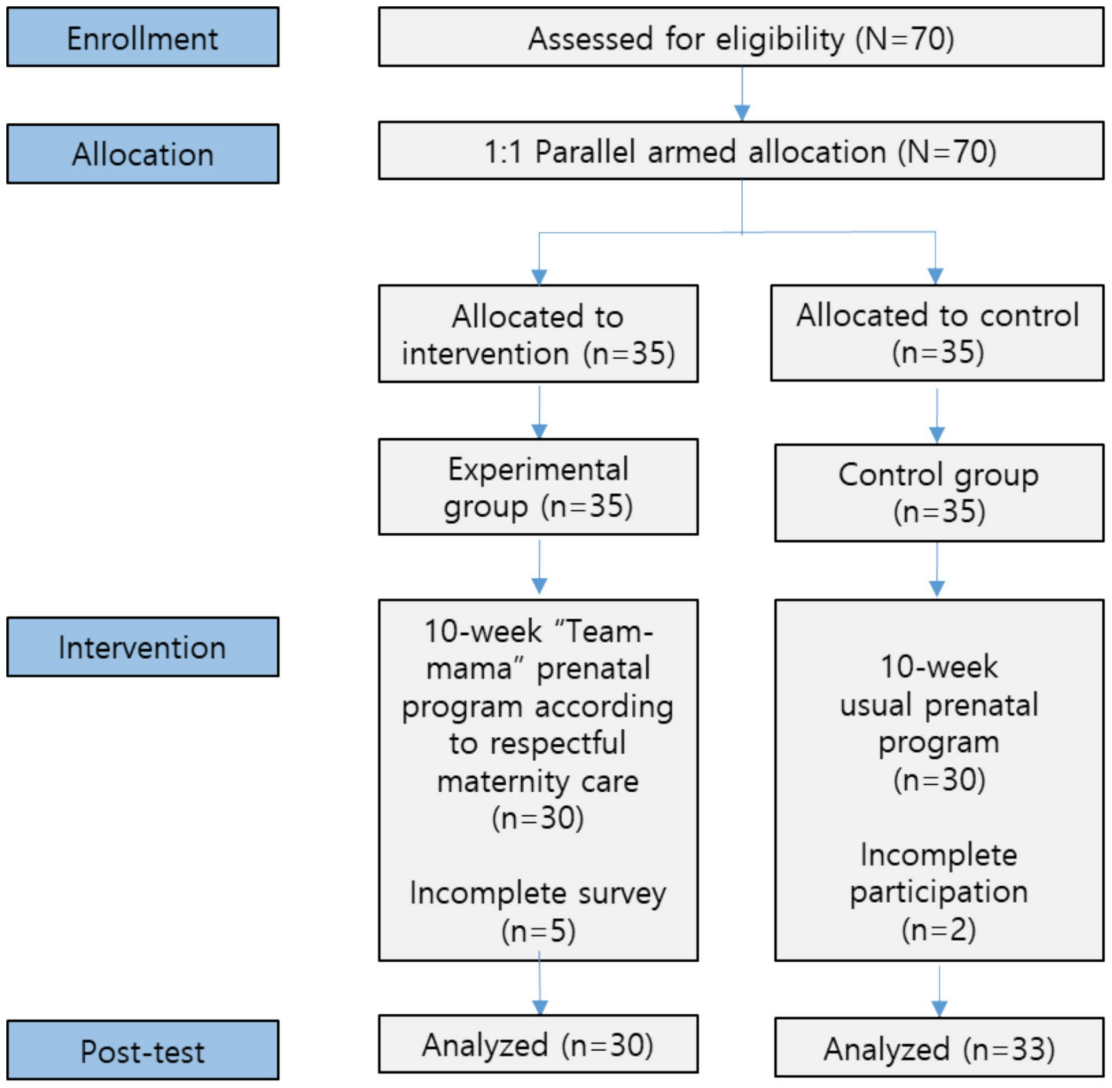
Flow diagram of the research process.

The sample size was determined based on an effect size (f) of 0.65 (14), a power of 0.80, a 1:1 allocation ratio, and a significance level of 0.05 for a 2-tailed *t*-test comparing 2 independent means, as calculated using the G*Power program (19). To account for potential dropouts, we aimed to enroll a total of 60 participants, with 30 in the experimental group and 30 in the control group. Anticipating a 15% dropout rate, we recruited 70 participants, with 35 in each group. In the control group, 2 participants did not participate in the entire program. In the experimental group, five failed to complete the survey (Figure 2).

### Ethical considerations

This study was approved by the Institutional Review Board of the researcher’s university (HIRB-2023–021). All measurement scales used in this study were employed with permission from the original authors. Participants were informed that they had the right to withdraw from the training, refuse to answer survey questions at any point, and revoke their consent. The researchers provided a detailed study description and obtained written consent for both participations in the program and the subsequent survey. To protect confidentiality, personal information was anonymized and encoded, ensuring that individuals could not be identified in the computerized data used for survey analysis.

### Data analysis

The data were analyzed using the SPSS/WIN 26.0 program. The obstetric characteristics, maternal outcomes, and neonatal birth outcomes of the participants were analyzed in terms of frequency, percentage, mean, and standard deviation. Homogeneity was tested between the 2 groups using the *t*-test and chi-square test. The differences between the 2 groups were examined using the *t*-test after assessing normality using the Shapiro–Wilk test.

## Results

### Homogeneity between the two groups

There were no statistically significant differences in the mean age (t = −1.73, *p* = 0.087), gravidity (t = 0.34, *p* = 0.731), parity (t = −1.17, *p* = 0.732), number of children (t = −0.87, *p* = 0.387), monthly income (t = −1.65, *p* = 0.104), employment status (*χ*^2^ = 0.51, *p* = 0.479), and education level (*χ*^2^ = 4.46, *p* = 0.215) between the 2 groups (Table 2).

**Table 2:** Analysis of homogeneity between the experimental and control groups (*N* = 63).

Characteristics	Control group (*n* = 33)	Experimental group (*n* = 30)	t/*χ*^2^	*p*
	*n* (%)/M (SD)	*n* (%)/M (SD)		
Age (years)	33.79 (4.54)	35.73 (4.31)	−1.73	0.087
Gravity	2.18 (0.88)	2.10 (0.99)	0.34	0.731
Parity	1.64 (0.74)	1.87 (0.81)	−1.17	0.732
Number of children	1.70 (0.72)	1.87 (0.81)	−0.87	0.387
Monthly income^§^	307.88 (156.40)	500.67 (650.67)	−1.65	0.104
Employment status				
Yes	15	11	0.51	0.479
No	18	19		
Education				
Middle school	2	0	4.46	0.215
High school	7	3		
College	21	21		
Graduate	3	6		

^§Korean dollar Won. M, mean; SD, standard deviation.^

### Effects on maternal birth outcomes

The experimental group exhibited lower rates of episiotomy, oxytocin augmentation, epidural anesthesia, and analgesics than the control group. Eleven participants (42.4%) in the control group had episiotomies, compared to none (0.0%) in the experimental group (*χ*^2^ = 16.36, *p* < 0.001). Eleven participants (33.3%) in the control group received oxytocin augmentation, compared to one participant (3.0%) in the experimental group (*χ*^2^ = 10.49, *p* = 0.005). Seven participants (21.2%) in the control group received epidural anesthesia, compared to none (0.0%) in the experimental group (*χ*^2^ = 7.15, *p* = 0.007). Fourteen participants (42.4%) in the control group used analgesics, compared to none (0.0%) in the experimental group (*χ*^2^ = 27.63, *p* < 0.001). Consequently, hypothesis 1 was fully supported (Table 3).

**Table 3:** Effects of “Team-mamas” continuity of midwifery care program on birth outcomes between groups (*N* = 63).

Variables		Control group (*n* = 33)	Experimental group (*n* = 30)	t/*χ*^2^	*p*
		*n* (%)/M (SD)	*n* (%)/M (SD)		
Neonate’s birth weight (g)		3.17 (0.45)	3.26 (0.32)	−0.95	0.346
Episiotomy	Yes No	14 19	0 30	16.36	<0.001
Oxytocin augmentation	Yes No Unknown	11 21 1	1 29 0	10.49	0.005
Epidural anesthesia	Yes No	7 26	0 30	7.15	0.007
Analgesics	Yes No Unknown	14 12 7	0 30 0	27.63	<0.001
Feeding at 1 week after birth	Breast Bottle Mixed	3 1 29	12 0 18	8.85	0.012
Feeding at 4 weeks after birth	Breast Bottle Mixed	4 0 29	14 1 15	10.89	0.004

^§Fisher’s exact test. M, mean; SD, standard deviation.^

### Effects on neonatal birth outcomes

The experimental group had a higher rate of breastfeeding than the control group. The mean weight of neonates in the experimental group was 3.26 kg (SD = 0.32), while the control group’s mean weight was lower at 3.17 kg (SD = 0.45). This difference was not statistically significant (t = −0.95, *p* = 0.346). One week after birth, three infants (9.1%) in the control group were breastfed, compared to 12 infants (40.0%) in the experimental group (*χ*^2^ = 8.85, *p* = 0.012). At 4 weeks postpartum, four infants (12.1%) in the control group were breastfed, compared to 14 infants (46.7%) breastfed in the experimental group (*χ*^2^ = 10.89, *p* = 0.004). Therefore, hypothesis 2 was partly supported (Table 3).

### Effects on birth satisfaction, birth experience, and maternal function

The experimental group participating exhibited higher birth satisfaction and birth experience than the control group. The experimental group reported higher overall birth satisfaction (M = 40.83, SD = 4.13) compared to the control group (M = 33.43, SD = 4.66) (t = −6.51, *p* < 0.001). This was reflected in the subcategories, which included the quality of care provision (t = −5.34, *p* < 0.001), women’s personal attributes (t = −2.24, *p* = 0.029), and stress experienced during labor (t = −6.67, *p* < 0.001).

The total score for birth experience was higher in the experimental group (M = 55.83, SD = 0.46) compared to the control group (M = 54.07, SD = 2.34) (t = −4.05, *p* < 0.001). The subcategories showed the following results: family support (t = −4.15, *p < 0*.001), personal care (t = −2.63, *p = 0*.011), affective empowerment (t = −2.16, *p = 0*.035), and information provision (t = −2.01, *p = 0*.049).

There were no statistically significant differences in total maternal function between the control group (M = 87.90, SD = 11.84) and the experimental group (M = 94.47, SD = 15.32) (t = −1.84, *p = 0*.067). The results for the subcategories were as follows: self-care (t = −0.75, *p =* 0.453), infant care (t = −0.62, *p = 0*.532), mother–child interaction (t = −0.79, *p = 0*.428), psychological wellbeing (t = −2.44, *p = 0*.018), social support (t = −0.78, *p = 0*.435), management (t = −1.07, *p = 0*.286), and adjustment (t = −0.87, *p = 0*.386). Consequently, hypothesis 3 was partially supported (Table 4).

**Table 4:** Effects of “Team-mamas” continuity of midwifery care program on birth satisfaction, experience, and maternal function between groups (*N* = 63).

Variables	Control group (*n* = 33)	Experimental group (*n* = 30)	*t*	*p*
	*n* (%)/M (SD)	*n* (%)/M (SD)		
Total birth satisfaction	33.43 (4.66)	40.83 (4.13)	−6.51	<0.001
Quality of care provision	14.13 (2.37)	17.30 (2.21)	−5.34	<0.001
Women’s personal attributes	4.47 (1.30)	5.30 (1.55)	−2.24	0.029
Stress experienced during labor	14.83 (2.35)	18.23 (1.50)	−6.67	<0.001
Total birth experience	54.07 (2.34)	55.83 (0.46)	−4.05	<0.001
Family support	19.40 (0.72)	19.97 (0.18)	−4.15	<0.001
Personal care	15.47 (0.93)	15.93 (0.25)	−2.63	0.011
Affective empowerment	9.50 (1.16)	9.97 (0.18)	−2.16	0.035
Information provision	9.70 (0.70)	9.97 (0.18)	−2.01	0.049
Total maternal function	87.90 (11.84)	94.47 (15.32)	−1.84	0.067
Self-care	11.63 (3.25)	12.33 (3.88)	−0.75	0.453
Infant care	10.23 (1.45)	10.50 (1.81)	−0.62	0.532
Mother–child interaction	13.90 (2.52)	14.43 (2.64)	−0.79	0.428
Psychological wellbeing	40.07 (6.59)	44.90 (8.60)	−2.44	0.018
Social support	14.33 (2.26)	14.87 (2.94)	−0.78	0.435
Management	25.17 (4.10)	26.53 (5.61)	−1.07	0.286
Adjustment	9.57 (1.40)	9.93 (1.81)	−0.87	0.386

M, mean; SD, standard deviation.

## Discussion

This study implemented the “Team-Mamas” CMC intervention that was provided throughout the antenatal, labor, and postpartum periods. This midwife-led program resulted in positive and safe delivery outcomes for mothers, highlighting the significance of offering high-quality maternity care. The CMC program improved maternal birth outcomes, as evidenced by reduced use of medications, decreased reliance on epidural anesthesia and narcotic analgesics, and a lower rate of perineal incisions, while also enhancing the birth experience and maternal satisfaction. Additionally, this study reinforced the role of midwives by demonstrating that CMC was associated with improved outcomes for both mothers and newborns (7).

South Korea’s total fertility rate was 0.72 in 2023, the lowest among Organization for Economic Co-operation and Development (OECD) countries and half the average rate for OECD nations. This low fertility rate reflects the challenges associated with childbirth and parenting (20). In a society facing a declining birthrate, the demand for high-quality maternity care becomes increasingly critical. Face-to-face antenatal care enhances physical and mental wellbeing and promotes a positive childbirth experience by ensuring women feel listened to and actively involved in clinical decision-making (21). Since the onset of the COVID-19 pandemic, opportunities for antenatal care have been limited due to personalization and initiatives involving internet-based education (22). The lack of childbirth education hinders expectant mothers from gaining the necessary knowledge, skills, and attitudes for proper health care (22). Consequently, many turn to self-help groups like “mom-cafés” or YouTube videos from non-professionals for information, which further impedes access to quality prenatal care (23).

The CMC intervention named “Team-Mamas” provided midwife-led education, counseling, care for women throughout childbirth. It aimed to reduce medical interventions and empower mothers to actively choose alternative birthing options, such as natural childbirth. Midwifery care has become the standard in numerous countries (24). However, in Korea, where 99.5% of births take place in hospitals in 2022 (20), midwives strive to facilitate natural births, which are distinct from typical hospital deliveries. This study supports midwives as the preferred professionals for childbearing women, offering continuous, holistic care from the prenatal to postpartum period—beyond a focus on merely operational or medicalized birth—within the context of a highly medicalized Korean society. Consistent with the maternal birth outcomes observed in this study, midwife-led deliveries have been associated with lower rates of cesarean sections, perineal incisions, and epidural anesthesia. Additionally, these deliveries have shown a decrease in neonatal intensive care unit admissions and an increase in the number of normal vaginal births (25).

Regarding infant birth outcomes, the CMC intervention using the RMC framework found that women who received midwife-led care had significantly higher breastfeeding rates at 1 week and at 4 weeks. A previous study reported that a higher proportion of women in the midwife-led care group (67%) were breastfeeding compared to those receiving standard care (46%) (26). Additionally, a meta-analysis showed that midwife-led care increased the likelihood of early initiation of exclusive breastfeeding (odds ratio, 1.88; 95% CI, 1.00, 2.77) (4). However, neonatal birth weight was not significantly affected by midwife-led care in a previous study, which is consistent with the findings of the current study. This contrasts with other research that identified midwife-led care as the gold standard, reporting its association with a reduction in preterm births (3, 4). Furthermore, midwife-led care did not appear to influence gestational age, suggesting that it may not have an effect on preterm birth or newborn weight (4). Further research is needed to clarify these discrepancies and better understand the specific conditions under which CMC influences birth outcomes.

Similar to the findings of this study, which indicate high labor satisfaction and positive labor experiences, continuous care provided by a midwife can support and assist pregnant women throughout childbirth. This care helps to alleviate pain and reduce anxiety and fear, leading to safer deliveries (14). In contrast, disrespectful and abusive care can result in adverse health outcomes, including psychological consequences such as post-traumatic stress and diminished trust in healthcare systems. The CMC program influenced stress relief, an empowering affective experience, and psychological wellbeing, which in turn affected birth satisfaction, experience, and maternal function. However, hospital deliveries continue to face issues related to respect, autonomy, dignity, privacy, and confidentiality in maternity care (8).

The findings of this study suggest that integrating the CMC model into healthcare policies can significantly improve the quality of maternity care. Health systems are encouraged to adopt CMC strategies to enhance maternal satisfaction and outcomes. Furthermore, these findings can support the creation of policies that prioritize woman-centered care, highlighting the importance of dignity, respect, and empowerment during childbirth. A move toward incorporating CMC into health policies may result in widespread improvements in maternal and neonatal health outcomes, as well as increased participants’ satisfaction throughout healthcare systems.

This study has several limitations, as follows: It used a non-equivalent control group quasi-experimental post-test design, which may not provide the same level of evidence as a randomized controlled trial. With only 65 participants, this limitation is particularly relevant for outcomes where no statistically significant differences were observed, as it is possible that true effects were present but not detected due to limited statistical power. The absence of blinding when allocating participants to the experimental and control groups could have introduced bias. No effects on maternal function were found because the “Team-Mamas” CMC program focused on pregnancy and birth care, not the postpartum period. The companion program primarily focused on pregnancy and birth care, with less attention given to the postpartum period, which may impact the comprehensiveness of the results.

## Conclusion

The study concluded that CMC using the RMC framework had a positive impact on women’s childbirth experiences by enhancing their satisfaction with intrapartum care. The findings further demonstrated the effectiveness of the CMC model in elevating the overall quality of maternity care. The results underscore the importance of integrating the CMC approach into healthcare systems to foster positive birth experiences. Adoption of this model holds significant potential to improve maternal health outcomes and increase satisfaction with maternity services.

## Data Availability

The raw data supporting the conclusions of this article will be made available by the authors, without undue reservation.
